# Quality management of head and neck patient treatments using statistical process control techniques

**DOI:** 10.1007/s13246-024-01469-y

**Published:** 2024-10-15

**Authors:** Michael J. Sandford, Jared G. Steel, Josie R. Goodworth, Patrick J. Lodge

**Affiliations:** 1 Mid North Coast Cancer Institute, Mid North Coast Local Health District Coffs Harbour Health Campus, Coffs Harbour, NSW 2450 Australia; 2 NSW Health, Sydney, NSW 2065 Australia

**Keywords:** Quality management, Statistical process control, Head & Neck, Rigid registration

## Abstract

The treatment, planning, simulation, and setup of radiotherapy patients contain many processes subject to errors involving both staff and equipment. Cone-beam-CT (CBCT) provides a final check of patient positioning and corrections based on this can be made prior to treatment delivery. Statistical Process Control (SPC) techniques are used in various industries for quality management and error mitigation. The utility of SPC techniques to monitor process and equipment changes in our Head and Neck patient treatments was assessed by application to CBCT results from a quality-focused longitudinal study. Individuals and moving range (XmR) as well as exponentially-weighted moving average (EWMA) techniques were explored. The SPC techniques were sensitive to process changes and trends over the 12 years of data collected. A reduction in the random component of patient setup errors needing correction was observed. Systematic components of error remained more stable. An uptick in both datasets was observed correlating with the COVID-19 pandemic. Process control limits for use in prospective process monitoring were established. Challenges that arose from using SPC techniques in a retrospective study are outlined.

## Introduction

Stabilisation is important in Head and Neck (H&N) Radiotherapy due to the relative proximity of PTVs and OARs. Patients receiving H&N radiotherapy at Mid North Coast Cancer Institute (MNCCI) are treated using Volumetric Modulated Arc Therapy (VMAT) to achieve conformal dose to complex targets while sparing healthy tissue [1]. Stabilisation equipment, simulation, and planning and treatment processes are all potential sources of error or quality change [2]. Thus, a level of monitoring is essential to ensure continuing benefit to patients. The use of statistical process control (SPC) methods in radiotherapy has become more prevalent in recent years [3]. The purpose of this longitudinal study was to assess the utility of statistical process control (SPC) methods for monitoring the process changes and establish process limits for prospective process control.

Initially, process and equipment changes were monitored by reviewing systematic and random errors from shift data as described by van Herk [2]. However, SPC methods used in other departmental domains based on published literature [4, 5], [6, 7] were readily adapted. Others have used SPC to review a similar cohort of patients, demonstrating appropriate sensitivity to process changes typical for a radiotherapy department, albeit using different technology [4, 8].

We report on the results of this study, using the combination of technology and processes particular to our department.

## Methods

### Patient selection

Patients were required to be able to lay flat while being fitted with a thermoplastic mask, vacuum bag, and indexed knee stabilisation. Multiple brands were used as described in Table 1. Patients who required variations to standard fitting protocol were excluded from this study to minimise extra study variables.

**Table 1:** RT system process changes relevant to H&N patient treatments between 2010 and 2023. Changes are codified for viewing in trend charts

Date	Code	Process change	Reason / notes
2010	A	H&N shims	Introduced to account for any mask shrinkage post-simulation and for patient comfort.
2014 – Jan	B	Daily CBCT	IGRT protocol change requiring daily CBCT for all H&N patients. VMAT techniques were introduced.
2014 – Jun	C	H&N vac-bag frame	Introduced to assist with shaping the vac-bags.
2015 – Jan	D	Localisation Trend Review (LTR)	Tolerances changed from 3 mm in a unilateral direction over 3 consecutive days to 1 cm.
2015 – May	E	Staff Education	All staff were given additional training in H&N simulation
2015 – Sep	F	Elekta Couch Move Assist (CMA)	New technology introduced.
2018 – Jan	G	Vac-bags changed	From Civco to Klarity 3-litre H&N vacuum bags.
2018 – Mar	H	Mask heating method	Waterbath replaced Klarity Airflow Oven [8].
2020 – Mar	I	Staffing and COVID measures	Short-staffing, high staff turn-over, and high portion of junior staff. COVID measures introduced, including non-rotating CT Simulation RT roles [9, 10].
2022 – Jun	J	Staffing	Staffing levels improved, turnover reduced, new grads trained.

### Clinical processes

Patients were scanned using Siemens computed tomography (CT) scanners (Siemens Healthineers, Erlangen, Germany). Images were exported to the treatment planning system (TPS) for structure delineation and planning. Patients were treated with Elekta linear accelerators equipped with X-ray Volumetric Imaging (XVI) systems (Elekta, Crawley, UK). Daily pre-treatment verification involved assessing patient deformation via either the cone-beam CT or 2DkV portal imaging with the planning CT. The resulting translations were recorded in Mosaiq. Various quality improvement initiatives resulted in both procedural and equipment changes as detailed in Table 1.

### Data collection and analysis

Data was extracted from Mosaiq. Analysis was performed within MS Excel and online statistics tools [9, 10].

### Determination of quality metrics for rigid registration

Rigid registration data for all patient fractions and courses was analysed for systematic and random components as per Moore et al. [8] with the scope reduced to translations only. The data was bony image-matched, and the XVI system reported the shifts directly. The shifts for each fraction were converted into a 3D registration vector *R*_*f*_, Eq. 1. The systematic component of the registration, *R*_*syst*_, over the course was determined as the mean geometric vector length over all fractions *f*, Eq. 2.

1 $${R}_{f}=\sqrt{{{(R}_{f,lat})}^{2}+{{(R}_{f,long})}^{2}+{{(R}_{f,vert})}^{2}}$$

2 $${R}_{syst}={\sum\:}_{f=1}^{F}\frac{{R}_{f}}{F}$$

The random component of the registration over the course, *R*_*rand*_, was determined as the vector sum of the standard deviations in each direction, Eqn’s 3–5.

3 $${SD}_{k}=\sqrt{\frac{1}{F-1}{\sum\:}_{f=1}^{F}{({R}_{f,k}-{m}_{f,k})}^{2}}$$

where

4 $${m}_{f,k}={\sum\:}_{f=1}^{F}\frac{{R}_{f,k}}{F}$$

5 $${R}_{rand}=\sqrt{{{(SD}_{lat})}^{2}+{{(SD}_{long})}^{2}+{{(SD}_{vert})}^{2}}$$

### Determination of statistical process control parameters

The rigid registration metrics, *R*_*syst*_ and *R*_*rand*_ formed the quality metrics for further assessment using SPC [8, 11], [12]. Course data were sorted by start date, and the process control limits (UCL, LCL) were determined by the formulas for individuals and moving ranges [8, 11, 12].

6 $$LCL,\:UCL=\underset{\_}{R}\pm\:3/{d}_{n}\cdot\:\frac{{\sum\:}_{i=2}^{t}\left|{R}_{i}-{R}_{i-1}\right|}{t-1}$$

*where* $$\:\:\:{d}_{n}=1.128$$

Exponentially weighted moving range (EWMA) smoothing was applied to the data to help identify process trends [13]. Here:

7 $${E}_{i}=\lambda\:{R}_{i}+\left(1-\lambda\:\right){E}_{i-1}$$

and

8 $$LCL,\:UCL=\underset{\_}{R}\pm\:L{\sigma\:}_{D}\sqrt{\left(\frac{\lambda\:}{2-\lambda\:}\right)}\left|1-{\left(1-\lambda\:\right)}^{2i}\right|$$

Where *R*_*i*_ is the rigid registration quality metric (systematic or random) for fraction *i*. $\:\lambda\:$ is a constant between 0 and 1 which sets the depth of smoothing. $\:\underset{\_}{R}$ and $\:\sigma\:$ are the mean and standard deviation, and $\:L$ is a scaling factor used to moderate the process limits range [13]. A Shapiro-Wilk test was used to assess data normality and help confirm reasonable values for the smoothing parameters.

The smoothing was initially applied to the full dataset to help identify, along with known process changes, a period of relative process stability to form a reference period. Process control limits and EWMA smoothing were recalculated based on the reference period data.

### Correlation of systematic vs. random components

The EWMA systematic registration data was plotted against the EWMA random registration data with the pre-, post-, and reference period groups delineated. A correlation-coefficient was determined for each group.

### Assessment of process change significance

The random and systematic data was grouped into populations with or without each identified process change. This was based on information associated with the patient data or using the date of the process change. For the latter, it was assumed that the introduction was effectively instant with respect to the resolution of the timeline. The groups were compared using a two-sided test Mann Whitney U test as no assumption was made regarding directionality. The null hypothesis was that the two populations were equal.

### Assessment of other factors

Previous research observed trends in patient weight loss [14, 15]. Therefore, the recorded patient weight measurements were analysed.

Given that the isocentre on bilateral H&N treatments remains along the patient’s midline removing the need for a lateral isocentre move (cf. uni-lateral), the H&N site localisation was also assessed for effect using a two-sided test Mann Whitney U test.

## Results

In total, 4441 fractions over 139 patients were assessed. The mean values for the 3D rigid registration vectors were 3.0 and 2.3 mm for the systematic and random components, respectively. The upper and lower process-control limits for the total dataset were 5.1–0.9 mm, and 3.7–0.9 mm; for the systematic and random components, respectively.

For the systematic components, a Shapiro-Wilk test showed statistically significant non-normality, W(139) = 0.978, *p* = 0.023, however the effect size was very small (0.060) so normality can be assumed. For the random components, a Shapiro-Wilk test showed a small departure from normality, W(139) = 0.931, *p* < 0.001, with medium effect size (0.108). Given the systematic data result, and the tolerance of the EWMA function to a range of $\:\lambda\:$ and $\:L$ values [13], the factors used for the smoothing were $\:\lambda\:$ = 0.05 and $\:L$ = 2.49 for both datasets, and for consistency with the key reference paper [8].

**Fig. 1:**
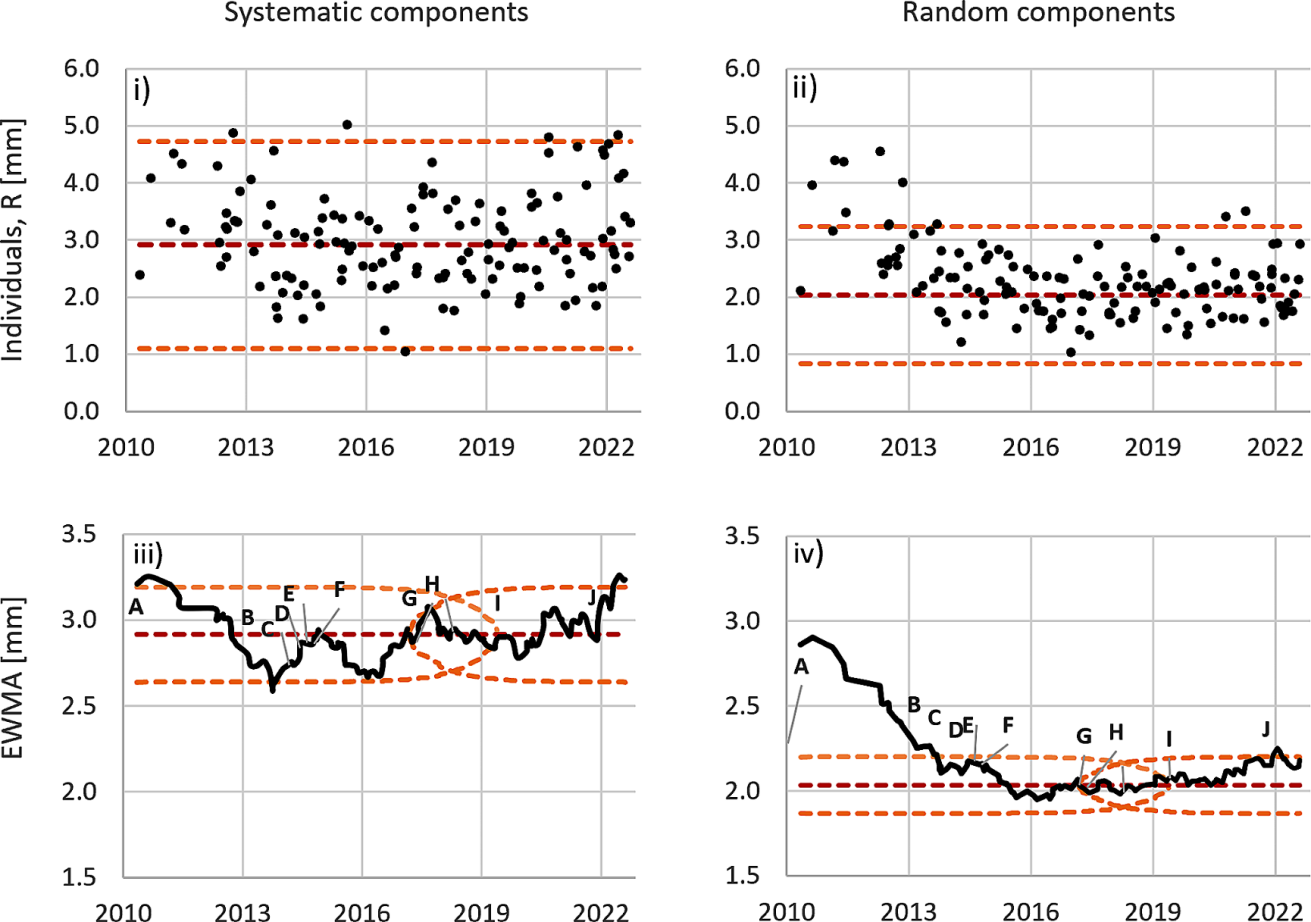
(i-iv) Time series of patient course quality metric data. Individuals of the systematic and random components are plotted with control limits calculated for the reference period only (i-ii). The EWMA is shown in (iii-iv), with control limits calculated for the reference period. Process changes are indicated by the letters A-J. Process means are shown (dashed red lines). Process control limits are indicated (dashed orange lines)

Using the smoothed data, the reference period was identified as 28 patient courses between Jan-2018 and Apr-2020. This related to process changes involving vac bags and the onset of COVID-19 and response measures. Mean values for the systematic and random quality metrics were 2.9 and 2.0 mm, respectively. The process limits for the systematic and random components in the reference period were 4.7–1.1 mm and 3.2–0.8 mm, respectively (see Fig. 1i-ii).

The EWMA charts were reconstructed, including process-control limits, using data from the reference period (see Fig. 1 iii-iv). For the systematic data, there was an early trend down from 3.3 mm to 2.7 mm, with a minima coinciding to the introduction of vac bag frames, and then a variable but upward trend (mean = 2.9 mm). The upper and lower EWMA process limits stabilised at 3.2 and 2.6 mm in the reference period, and this was mirrored in the retrospective analysis. The random component data portrayed a clear trend down starting at 2.9 mm towards a mean of 2.0 mm during the reference period. An upward trend was observed from dates relating to staffing issues and COVID measures. The retrospective EWMA period 1 data showed upper and lower process limits stabilising at 2.2 and 1.9 mm, which were again mirrored in the retrospective projection. Overall, the biggest improvement – reductions in process mean and variance over time – was observed in the random component quality metric. After sitting near the mean in the reference period, both data sets trended up and exceeded the process-based control limits in the later months.

**Fig. 2:**
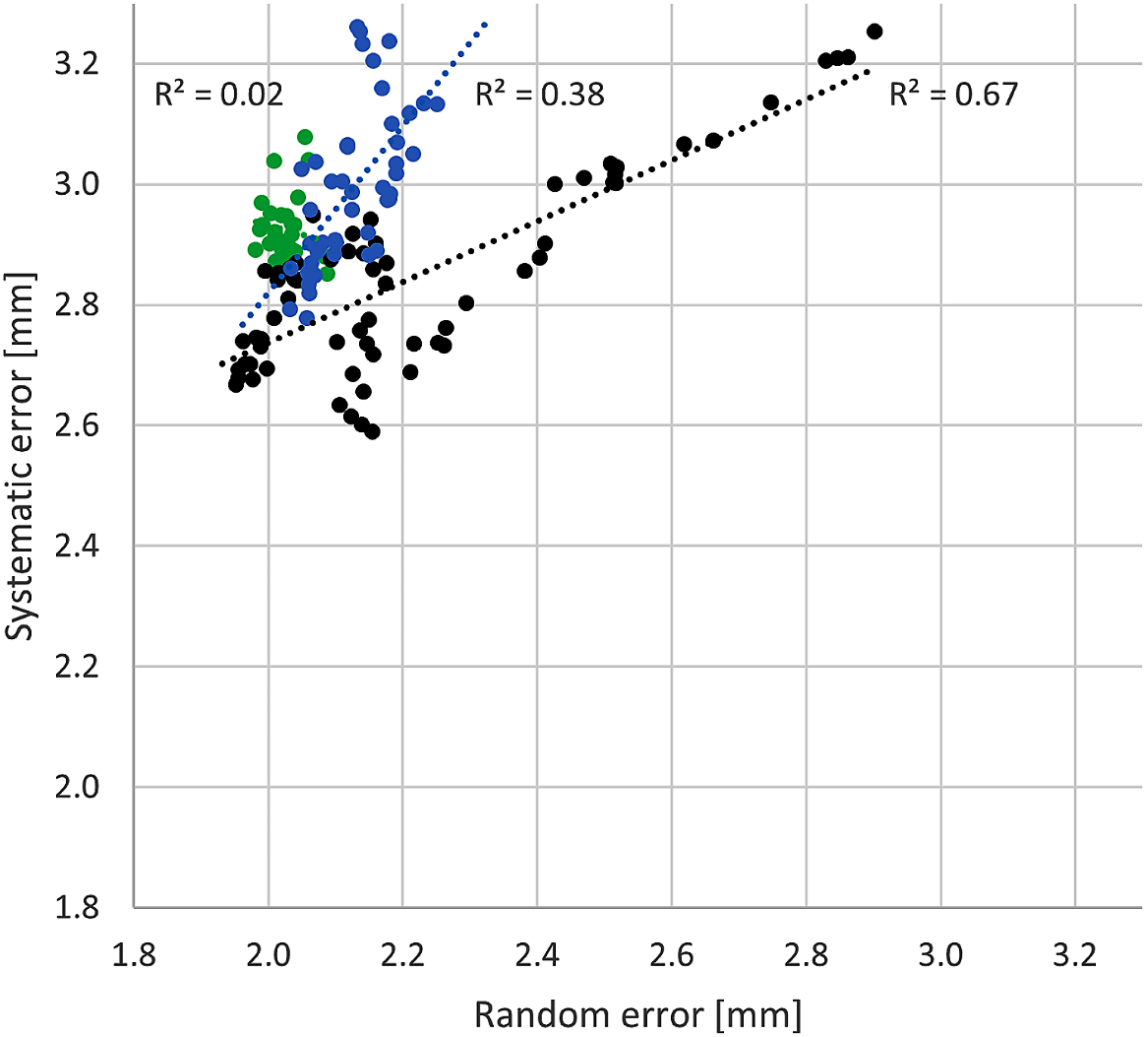
Correlation plot of systematic and random components of setup error. Time period prior to the review period is indicated in black. The reference period data is indicated in green. The post-review period data is indicated in blue

The correlation plot showed distinct regions for each grouping. Between the pre-reference period data vs. the reference and post-reference period data there was a moderate shift in the y-axis corresponding to systematic components, and a more distinct shift in the x-axis corresponding to random components (Fig. 2).

Results of the subgroup analysis is shown in Table 2. There was a difference in significance levels between the systematic and random components. There was a trend whereby significance is observed for the earlier process changes and not for later changes. The exception was in the systematic data which showed a significant change relating to staffing in later months.

**Table 2:** Results of sub-group analysis via Mann Whitney U testing (2-tailed, unpaired)

Process change	Date		N	Systematic [mm]				Random [mm]			
				Mean	CI low	CI high	P-val	Mean	CI low	CI high	P-val
Shims	Nov 2010	Prior	0								
		Post	139								
Daily CBCT	Jan 2014	Without	18	3.5	3.2	3.9	0.002*	3.2	2.8	3.6	0.000*
		With	121	2.9	2.8	3.1		2.1	2.1	2.2	
Vac-bag frame	Jul 2014	Prior	22	3.4	3.1	3.8	0.004*	3.1	2.7	3.4	0.000*
		Post	117	2.9	2.8	3.1		2.1	2.0	2.2	
Trend review	Jan 2015	Prior	31	3.2	2.9	3.5	0.214	2.9	2.6	3.1	0.000*
		Post	108	2.9	2.8	3.1		2.1	2.0	2.2	
Staff education	May 2015	Prior	35	3.1	2.8	3.4	0.664	2.8	2.5	3.0	0.000*
		Post	104	3.0	2.8	3.1		2.1	2.0	2.2	
Elekta CMA	Sep 2015	Prior	37	3.0	2.7	3.3	0.832	2.7	2.5	3.0	0.000*
		Post	102	3.0	2.8	3.1		2.1	2.0	2.2	
New vac-bags	Jan 2018	Prior	67	2.9	2.7	3.1	0.475	2.5	2.3	2.6	0.004*
		Post	72	3.1	2.9	3.2		2.1	2.0	2.2	
CT oven	Apr 2018	Prior	70	2.9	2.7	3.1	0.391	2.4	2.2	2.6	0.019*
		Post	69	3.1	2.9	3.3		2.1	2.0	2.2	
Staffing and COVID	Apr 2020	Prior	92	2.9	2.8	3.1	0.210	2.3	2.2	2.5	0.185
		Post	47	3.1	2.9	3.4		2.2	2.0	2.3	
Staffing	Sep-2022	Prior	124	2.9	2.8	3.1	0.022*	2.3	2.2	2.4	0.932
		Post	15	3.5	3.0	4.0		2.2	2.0	2.5	

*indicates significance to p ≤ 0.05

**Fig. 3:**
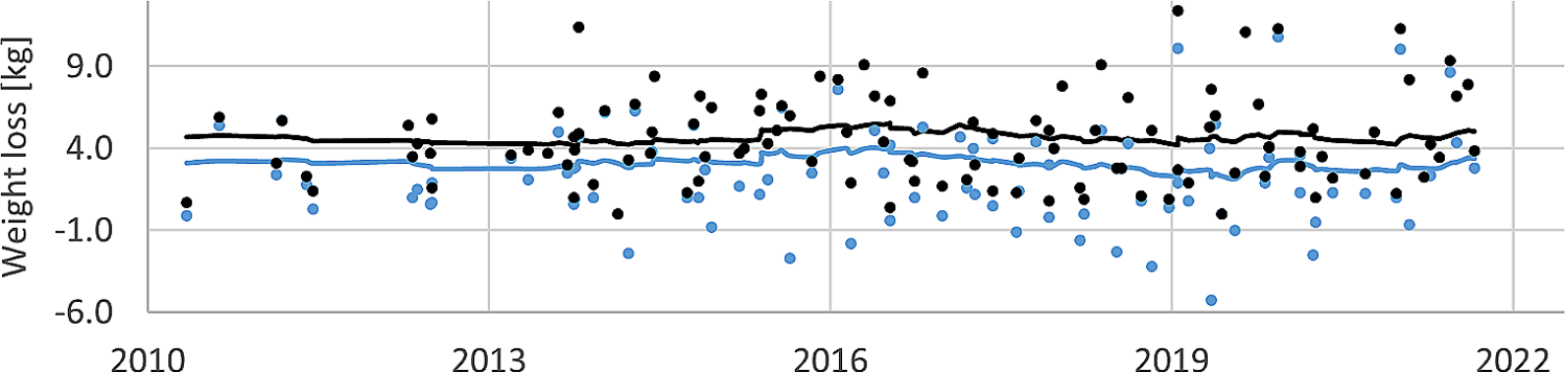
Time-series of weight loss over each patient course. Blue designates the difference between last and first course weight readings, black designates the difference between maximum and minimum weight readings. Red dots indicate the patients (*n* = 2) where a replan was triggered. The trend via EMWA smoothing is shown as solid lines

Patient weight changes, either by last-first or max-min difference, was shown to be varied with no obvious trend. On average patients lost 3.1 ± 3.4 kg (1. SD) between the last and first course recorded (Fig. 3). A comparison of Bi-lateral vs. Uni-lateral setup types showed no significant difference in the systematic or random components, *p* = 0.6055, and *p* = 0.8749 respectively.

## Discussion

The study focused on process and equipment changes to determine systematic and random components of registration shifts, and any trends therein. While we could have focussed on each translation dimension independently as others have done [7], we considered the route taken by Moore et al. [8] regarding combining these towards determination of random and systematic components to be more informative for process management. Obviously, it may be pertinent to investigate trends in individual dimensions if significant process deviations are detected.

A typical approach for establishing process limits is to use the first *n* results, as per Li et al. [7], however this may not be appropriate for retrospective assessment of a process that is improving over time. For this we again followed Moore et al. [8] to identify a reference period allowing the smoothing function in the EWMA assessment to be decoupled from the earliest data, which would otherwise be distorted by the initial trend. We identified a period where the data indicated the process was below the overall mean in both the systematic and random datasets and informed by discussion with clinical staff. However, there are other periods that may have satisfied these criteria which may have resulted in different process limits.

With the reference period established, the EWMA data show an overall improvement in the random error (Fig. 1, iv), and similar to Moore et al. [8] this is reflected in the correlation plot with the review and post-review period data consolidated and shifted distinctly left in the x-axis.

There are many factors incorporated in the trends observed. Anatomical changes including tumour regression or progression, weight loss, and edema can potentially contribute to the recorded shifts [16]. Total weight loss is a proxy for soft tissue loss impacting mask and vac-bag fit, with replanning indicated at certain tolerances. However, no systematic change in weight loss trends was observed, and only 6 patients were replanned due to contour changes. Nevertheless, due to the retrospective nature of the study, beyond total weight loss, other anatomical factors were not able to be fully controlled for.

Imaging protocols changed over the 12 years, e.g., moving from daily CBCT first 3 fractions then weekly (planar imaging other days), to a full course daily CBCT. A*d-hoc* variations to the protocol were included, e.g., on advice from the Radiation Oncologist; due to the PTV/OAR locations; or replanned with daily CBCT over the second phase. We observed significant improvements to both the systematic and random components of registrations shift around the time daily CBCT was introduced, however the change was not clean, confounding the observation.

Other protocol aspects are important to consider. For instance, 13 of the first 30 patients had Localisation Trend Reviews (LTRs) actioned, which re-baselines the patient’s setup. The protocol was tighter for this group. It is presumed that the shift values recorded were more conservative than for the remainder of the patients, however the data is blind to this. For the LTR actioned patients, a significant change in random components of registration shifts was observed.

From early 2020 numerous things occurred relating to the onset and response to the COVID-19 pandemic. Firstly, the department experienced a high turnover of staff, short-staffing, and had a high-portion of junior staff. Based on previous SARS experience [17, 18], infection control measures such as reduced staff rotation were implemented, including in CT. Impacts to treatment quality from infection controls and staff anxiety have been noted [19]. An increase in both systematic and random error over this time towards and exceeding the EWMA control limits was observed. RT managers’ report that staff level, churn, and experience profile returned to normal post-COVID. Notably the EWMA random components ticked back into control from late 2022, however the systematic components remain outside control limits. This may justify a closer look at the impact of staff levels and rotations to identify other causes, including human factors more broadly.

Similar to other reports [8], we found the process change significance correlated with the timing of introduction, suggesting that over time otherwise meaningful changes have diminishing returns on process improvement. However, this does not establish the relative significance of each process change in isolation, and changes that didn’t lead to an observed improvement in this study should still be considered in any quality improvement initiative. Further caution is needed for results with low *n*, which undermined the otherwise significant results with respect to systematic components. The random components carried results with valid significance under the Mann-Whitney U-test analysis. As noted by others [8], these limitations point to the obvious advantage of a prospective SPC approach where potentially significant process changes can be detected well before there is enough data to confirm it by other means, or where tracking the process over sufficient data points may accrue other confounding factors. Therefore, use of a planned approach where clinical changes are monitored prospectively, with process limits established early, is supported. We recommend considering process control techniques when commissioning new technologies and processes. While an assessment of the impact from specific changes is limited, a retrospective analysis nevertheless remains valuable to confirm overall process improvement and inform quality management expectations moving forward.

## Conclusion

A retrospective analysis of 139 H&N patient treatments was performed. The rigid registration of these patients improved overall, especially the random components of setup error. Numerous process changes appeared in combination to be significant towards this outcome, however attributing causality and determining the relative importance of each was confounded by the overlap of these interventions. SPC tools proved sensitive to process changes where descriptive statistics techniques were confounded. Process limits based on systematic and random components in the individual course 3D rigid registration space have been established, as well as via an EWMA smoothing technique, which can be used for monitoring the registration process of H&N patients prospectively.

## Data Availability

The deidentified data that support the findings of this study are available from the corresponding author, upon reasonable request.

## References

[1] Bhide S, Nutting C (2010) Recent advances in radiotherapy. BMC Med 8(1):25. 10.1186/1741-7015-8-25 10.1186/1741-7015-8-25 PMC2873246 20426851

[2] van Herk M (2004) Errors and margins in radiotherapy. Semin Radiat Oncol 14(1):52–64. 10.1053/j.semradonc.2003.10.003 10.1053/j.semradonc.2003.10.003 14752733

[3] Raveendran V, R GR, Bhasi APTS, R. CP, and Kinhikar RA (2023) Moving towards process-based radiotherapy quality assurance using statistical process control. Physica Med 112:102651. 10.1016/j.ejmp.2023.102651 10.1016/j.ejmp.2023.102651 37562233

[4] Pawlicki T, Whitaker M, Boyer AL (2005) Statistical process control for radiotherapy quality assurance. Med Phy 32(9):2777–2786. 10.1118/1.2001209 10.1118/1.2001209 16266091

[5] Miften M et al (2018) Tolerance limits and methodologies for IMRT measurement-based verification QA: Recommendations of AAPM Task Group No. 218. Med Phy 45(4):e53–e83, 10.1002/mp.12810 10.1002/mp.12810 29443390

[6] NSW Government - Clinical Excellence Commission Control Charts. Clinical Excellence Commission - Control Charts. [Online]. Available: https://www.cec.health.nsw.gov.au/CEC-Academy/quality-improvement-tools/control-charts. Accessed 07 Sept 2023.

[7] Li Z et al (2021) Performance assessment of surface-guided radiation therapy and patient setup in head-and-neck and breast cancer patients based on statistical process control. Physica Med 89:243–249, 10.1016/j.ejmp.2021.08.007 10.1016/j.ejmp.2021.08.007 34428608

[8] Moore SJ, Herst PM, Louwe RJW (2018) Review of the patient positioning reproducibility in head-and-neck radiotherapy using Statistical Process Control. Radiotherapy Oncol 127(2):183–189. 10.1016/j.radonc.2018.01.006 10.1016/j.radonc.2018.01.006 29395288

[9] Statistics, Kingdom Statistics Kingdom. Statistics Kingdom. [Online]. Available: http://www.statskingdom.com. Accessed 07 Sept 2023.

[10] Microsoft, Corporation (2018) Microsoft Excel. [Online]. Available: https://office.microsoft.com/excel

[11] Wheeler DJ (2010) Individual Charts Done Right and Wrong. Quality Digest Feb. 01, 2010. [Online]. Available: https://www.qualitydigest.com/inside/twitter-ed/individual-charts-done-right-and-wrong.html. Accessed 17 Dec 2023.

[12] Wheeler DJ (2010) The Right and Wrong Ways of Computing Limits. Quality Digest Jan. 07, 2010. [Online]. Available: https://www.qualitydigest.com/inside/twitter-ed/individual-charts-done-right-and-wrong.html. Accessed 17 Dec 2023.

[13] Montgomery DC (2020) Introduction to statistical quality control, Eighth edition. Hoboken, NJ: John Wiley & Sons, Inc

[14] Cacicedo J et al (2013) A prospective analysis of factors that influence weight loss in patients undergoing radiotherapy. Chin J Cancer. 10.5732/cjc.013.10009 10.5732/cjc.013.10009 PMC3975186 24103791

[15] Ottosson S, Zackrisson B, Kjellén E, Nilsson P, Laurell G (2013) Weight loss in patients with head and neck cancer during and after conventional and accelerated radiotherapy. Acta Oncol 52(4):711–718. 10.3109/0284186X.2012.731524 10.3109/0284186X.2012.731524 PMC3622234 23106176

[16] Li H et al (2008) Comparison of 2D Radiographic Images and 3D Cone Beam Computed Tomography for Positioning Head-and-Neck Radiotherapy Patients. Int J Radiat Oncol Biol Phys 71(3):916–925, 10.1016/j.ijrobp.2008.01.008 10.1016/j.ijrobp.2008.01.008 18395358

[17] Tang J et al (2005) Patient satisfaction with doctor-patient interaction in a radiotherapy centre during the severe acute respiratory syndrome outbreak. Australas Radiol 49(4):304–311, 10.1111/j.1440-1673.2005.01467.x 10.1111/j.1440-1673.2005.01467.x PMC7185414 16026437

[18] ABC Coffs Coast (2020) Coronavirus threatens cancer sufferers, but SARS-style protocols can help, doctor says. ABC News, Mar. 24

[19] Mohindra P, Buckey CR, Chen S, Sio TT, Rong Y (2020) Radiation therapy considerations during the COVID-19 pandemic: literature review and expert opinions. J Appl Clin Med Phys 21(5):6–12. 10.1002/acm2.12898 10.1002/acm2.12898 PMC7286011 32324950

